# Da-Cheng-Qi decoction improves severe acute pancreatitis capillary leakage syndrome by regulating tight junction-associated proteins

**DOI:** 10.3389/fphar.2024.1138251

**Published:** 2024-04-19

**Authors:** Jiamin Tu, Yinping Jiang, Lei Tu, Yafeng Chen, Liyun Pan, Xinjuan Fan, Jiyun Tian, Jianguo Li, Xinji Wang, Honghao Fu, Bing Xu, Dianxu Feng

**Affiliations:** ^1^ Department of Proctology, University of Chinese Academy of Science Shen Zhen Hospital (GuangMing), ShenZhen, China; ^2^ Department of Chinese Medicine, University of Chinese Academy of Science Shen Zhen Hospital (GuangMing), ShenZhen, China; ^3^ Department of General Surgery, Putuo Hospital, Shanghai University of Traditional Chinese Medicine, Shanghai, China; ^4^ Department of Traditional Chinese Medicine, The First People’s Hospital of Changzhou, The Third Affiliated Hospital of Soochow University, Changzhou, China; ^5^ Department of Ophthalmology, Quzhou Traditional Chinese Medicine Hospital, Quzhou, China

**Keywords:** acute necrotizing pancreatitis, tight junction, Da-Cheng-Qi decoction, capillary endothelial barriers dysfunction, claudin, occludin, ZO-1, JAM-C

## Abstract

**Background and aims:**

To investigate mechanisms underlying the effects of Da-Cheng-Qi decoction (DCQD) on severe acute pancreatitis (SAP) capillary leakage syndrome.

**Methods:**

In this study, a SAP rat model was established using retrograde perfusion of 5% sodium taurocholate into the biliopancreatic duct. The study included three randomized groups: control, SAP (modeling), and DCQD (via gavage at 2 h pre-modeling and 2 and 4 h post-modeling). HPLC was used to analyzed major components of DCQD. Pathological changes and capillary permeability in the rat pancreatic tissues were examined. mRNA levels of claudin 5, occludin, zonula occludin-1 (ZO-1), and junctional adhesion molecules (JAM-C) were assessed using qRT-PCR. Tight junction-associated protein expression was evaluated using immunofluorescence and Western blot analyses. Human umbilical vein endothelial cells (HUVECs) were used to investigate the mechanism m of DCQD.

**Results:**

Serum levels of amylase, TNF–α, IL-1β, IL-2, and IL-6 were higher in the SAP group compared to the DCQD group (*p* < 0.05). DCQD treatment significantly attenuated rat pancreas damage (*p* < 0.05) and reduced tissue capillary permeability compared to the SAP group (*p* < 0.05). Claudin 5, occludin, and ZO-1 expression in the rat tissues was upregulated, but JAM-C was downregulated by DCQD treatment (*p* < 0.05). HUVEC permeability was improved by DCQD in a dose-time-dependent manner compared to the SAP group (*p* < 0.05). DCQD also upregulated claudin 5, occludin, and ZO-1 expression *in vitro* (*p* < 0.05).

**Conclusion:**

DCQD can improve capillary permeability in both *in vivo* and *in vitro* models of SAP by upregulating expression of claudin 5, occludin, and ZO-1, but not JAM-C.

## Introduction

Severe acute pancreatitis (SAP) is a common clinical abdominal emergency. SAP is associated with severe organ damage, bleeding, and inflammation that result in death in approximately 35%–50% of SAP cases (Chinese Pancreatic, 2021). Systemic inflammatory response syndrome (SIRS) and multiple organ dysfunction syndromes (MODS) often occur at an early stage of SAP, which could lead to vascular endothelial cell damage, capillary endothelial barrier function dysfunction, and capillary leakage syndrome (CLS) (Kylanpaa et al., 2010). CLS occurs when vascular permeability increases and vascular content leaks out from the damaged endothelial barrier, provoking vasospasm, blood stasis, tissue ischemia, and necrosis (Kylanpaa et al., 2010). Because severe capillary leakage is one of the important complications of SAP in association with MODS, prevention and treatment of capillary leakage are crucial for stopping disease process and reducing SAP-associated morbidity and mortality.

In SAP pathogenesis, capillary leakage is mainly caused by severe capillary endothelial barrier function impairment (Kylanpaa et al., 2010). The vascular endothelial barrier is a semi-selective permeability barrier consisting of a single layer of endothelial cells and a basement membrane in the inner wall of blood vessels (Sukriti et al., 2014). Under physiological conditions, components inside and outside the blood vessels can enter and exit the vasculature and tissues through the vascular endothelial barrier; therefore, the integrity and function of the barrier is essential for maintaining homeostasis in the organism. Acute pancreatitis commonly affects the pancreas as a target organ. Evaluating the permeability of the pancreas serves as an objective measure to assess endothelial barrier function (Sukriti et al., 2014). As such, vascular permeability-associated factors, such as tight junction (TJ)-associated proteins located at the outermost part of the capillary endothelial intercellular junction, are used as markers of vascular leak. TJ-associated proteins play an important role in maintaining endothelial cell barrier function, stabilizing capillary permeability, and maintaining capillary internal and external homeostasis (Otani and Furuse, 2020). The main TJ-associated proteins include three transmembrane proteins, claudins, occludins, and junctional adhesion molecules (JAMs), but the TJ also includes cytoplasmic proteins, such as zonula occludin (ZO). Occludin, ZO-1, and JAM-C can form a complex in the TJ that be detected in a co-precipitation assay. This complex is involved in the regulation of leukocyte-endothelial cell interactions that are associated with trans-endothelial infiltration of monocytes and microvascular permeability, both of which are involved in inflammatory diseases (Buckley and Turner, 2018; Heinemann and Schuetz, 2019). Some TJ-associated proteins are tissue and organ specific, such as claudin 5 (Schreibelt et al., 2007) and JAM-C (Mandell and Parkos, 2005), which are only expressed in vascular endothelial cells.

The traditional Chinese formula Da-Cheng-Qi decoction (DCQD) is composed of *Rheum palmatum L*. (Dahuang), *Magnolia henryi Dunn*. (Houpu), *Citrus aurantium L*. (Zhishi), and *NatriiSulphas* (Mangxiao) and has been applied to treat SAP effectively for decades in China (Xia et al., 2006). DCQD can ameliorate disease severity and complications of CLS. However, the mechanisms underlying its effects on CLS remain unclear. In our previous study (Xie et al., 2014), we found that DCQD treatment reduced capillary permeability and improved capillary barrier dysfunction in a SAP animal model, but we did not investigate the mechanism of action of DCQD. We hypothesized that expression of TJ-associated proteins, including claudin 5, occludin, ZO-1, and JAM-C, are altered in SAP. Here, we investigated the mechanism of DCQD in the treatment of SAP-associated CLS using a SAP animal model to test our hypothesis.

## Materials and methods

### Experimental animals

SPF healthy Sprague–Dawley rats (40 male, body weight 200 ± 20 g) were provided by SLAC Laboratory Animals Co. (China). All animal experiments were done in accordance with the Animal Ethics Committee Guidelines of the University of Chinese Academy of Science Shen Zhen Hospital (GuangMing) (NO. LL-KT-2019043) before initiation of the study. Rats were fasted 24 h before experiments but had free access to water.

### Cell culture

The Cell Bank of the Chinese Academy of Sciences provided human umbilical vein endothelial cells (HUVECs) (No# EA-hy926), which were cultured in DMEM high glucose complete medium supplemented with 10% fetal bovine serum (FBS), 100 U/mL penicillin, 100 ng/mL streptomycin, 10 mM glutamine, 1 ng/mL hydrocortisone, and 10 ng/mL epidermal growth factor. Cells were maintained in an incubator at 37°C with 5% CO_2_.

### Preparation of serum medium from SAP rats

To establish a rat model of SAP, experimental rats were treated with 5% sodium taurocholate (Sigma, United States) at a dose of 1 mL/kg body weight, via a slow biliopancreatic duct injection using a micro-infusion pump at a rate of 0.2 mL/min. Blood was taken from the abdominal aorta 12 h after treatment. Blood samples were placed at room temperature for 2 h, and the serum was obtained. The amylase (Amy) value was usually 3–5 times higher in the SAP rats compared to the normal rats. SAP rat serum was filtered through a 2 μm filter, followed by adding DMEM medium at a 10-fold dilution (i.e. 10% concentration).

### DCQD preparation

DCQD was prepared using freeze-dried drug powders provided by the School of Pharmacy, Shanghai University of Traditional Chinese Medicine. The preparation procedure was based on the Treaties on *Exogenous Febrile Disease*, with a formulation including 12 g of *Rheum palmatum L.*, 24 g of *M. henryi Dunn.*, 12 g of *C. aurantium L.,* and 9 g of *Natrii Sulphas*. Briefly, the raw herbs of *M. henryi Dunn.* and *C. aurantium L.* were soaked in 20 L potable water for 30 min, then boiling water for 20 min, followed by adding the raw herb of *Rheum palmatum L.*, keeping the water boiling slightly for 15 min. The liquid containing water-soluble components of the herbs was taken to dissolve another herb *NatriiSulphas.* After complete dissolution by vigorously stirring, the medicine-containing liquid was filtered through a 200-mesh sieve, and the insoluble residue was discarded. The filtered liquid was concentrated under reduced pressure (the temperature of the material did not exceed 55°C) to 2.7 L. The extract was further concentrated by adding 4.7 L of 95% medical grade alcohol, stirring well until the alcohol concentration was reduced to about 60%. After 24 h, the supernatant alcohol was recovered under reduced pressure (Li et al., 2012). The aqueous-alcoholic precipitate was freeze-dried to yield a solid dispersion (246 g of solid, yield: 9.11%).

### *In vitro* and *in vivo* intervention

For *in vitro* intervention, five groups of HUVECs were included: low DCQD dose group (LDG, 25 μg/mL), medium DCQD dose group (MDG, 50 μg/mL), high DCQD dose group (HDG, 100 μg/mL) (Li, 1999), Control group (CG), and SAP serum-treated model group (SG) (Zhong, 2010). Cells in each group were treated for 6, 12, and 24 h, respectively.

For *in vivo* intervention, 30 rats were randomly divided into three groups (*n* = 10 per group): control group (CG), SAP group (SG), and DCQD group (DG). The SAP rat model was established as mentioned above. The CG group received gentle turning of the pancreas, followed by suturing of the layers of the abdominal wall. The DCQD powder was dissolved in saline solution (88.56 mg/day/rat, 1 g solid dispersion containing 10.9756 g original DCQD drug). The SAP rats were treated with DCQD by oral gavage at 2 h before and at 2 and 4 h after modeling. The treatment dose of DCQD was determined based on the rat-human equivalent dose conversion factor (0.018), with 60 kg body weight for humans and 200 g body weight for rats.

### Sample collection and measurements

HUVECs were harvested at 6, 12, and 24 h after modeling. The cells were washed with PBS followed by isolation of RNA for qRT-PCR analysis.

All experimental rats were sacrificed 24 h after modeling and treatment. One hour before sacrifice, the experimental rats were injected with 2% Evan’s blue (EB; Sigma, United States) via the penile vein at a dose of 0.1 mL/100 g body weight. Once rats were sacrificed, blood was drawn from the aorta and the pancreas was immediately collected. After centrifuging at 3,000 rpm for 5 min, the supernatants of the blood samples were collected and aliquoted into Eppendorf tubes, which were stored at −20°C for subsequent analysis of serum cytokines. After washing with normal saline, tissue samples were divided into three equal portions for different experimental protocols: (1) formalin-fixed tissue sectioning for hematoxylin and eosin (H&E) staining, (2) tissue sectioning to measure EB as a marker of tissue capillary permeability (Saria and Lundberg, 1983), and (3) tissue storage in liquid nitrogen for subsequent qRT-PCR and Western blot analyses. Pathological scores were evaluated blindly by two independent pathologists using a Schmidt scoring system (Schmidt et al., 1992).

#### Cell proliferation assays

Cell viability was measured using a Cell Counting Kit-8 (CCK-8, Japan) according to the manufacturer’s instructions. Briefly, 1.5×10^4^ cells/well were seeded in a 96-well plate and cultured for 24 h, followed by treatment with different concentrations of DCQD: 1 mg/mL, 100 μg/mL, 10 μg/mL, 1 μg/mL, 100 ng/mL, 10 ng/mL, and 1 ng/mL. To visualize viability, 10 μL of the CCK-8 kit solution was added into each well at 6, 12, and 24 h after treatment. After incubation at 37°C for 30 min, the absorbance (450 nm) of each well was measured using a microplate reader.

## HPLC

Nine major components of DCQD were determined using HPLC analysis. Quality control samples were prepared to obtain the following plasma components for rheum emodin; 0.1884 mg/mL for chrysophanol; 0.0403 mg/mL for physcion; 0.421 mg/mL for naringin; 0.166 mg/mL for hesperidin, 0.646 mg/mL for magnolol, and 0.5127 mg/mL for honokiol. The samples were pretreated and detected in each analytical batch, except for samples one and two which were diluted 1-fold in ethanol for analysis. HPLC analysis was performed in the Engineering Research Center of Modern Preparation Technology of Shanghai University of Traditional Chinese Medicine (SHUTCM), Ministry of Education. The results of HPLC analysis of DCQD components were deposited in the Central Laboratory of Shanghai Putuo District Central Hospital (Shanghai, China).

### Measurement of serum cytokine levels

Serum levels of Amy were measured using a Cobas Integra 400 analyzer (Roche Diagnostics Systems, Switzerland). Serum levels of tumor necrosis factor-α (TNF-α), interleukin (IL)-1β, IL-2, and IL-6 were determined using ELISA kits (MultiSciences Co., LTD).

### Transendothelial electrical resistance (TER)

TER is a rapid, non-invasive method for quantifying barrier tissue integrity by measuring the electrical resistance across the tissue. Briefly, a 100 μL cell suspension of HUVECs (1×l0^5^ cells/mL) was seeded into the inner side of a Transwell cell insert (Corning, United States) (pore size 0.4 μm, growth area 0.3 cm^2^), followed by adding 300 μL of serum-free DMEM into the outer cell insert. When cell adhesion occurred, the medium was aspirated, followed by adding 200 μL of medium containing treatments to the inner well and 600 μL of serum-free DMEM to the outer well. The electrical resistance, also known as ohmic resistance (Ω), was measured using a resistograph device at 6, 12, and 24 h after modeling. Measurements were repeated three times in each chamber at different locations.

### FITC-dextran permeability test

The preparation for cell suspension of HUVECs (1×l0^5^ cells/mL) that were seeded into the Transwells was the same as the TER assay (described above). At 6, 12, and 24 h after modeling, 30 μL of 10 mg/mL FITC-dextran (Sigma, United States) was added into the cell insert with an extra incubation of 30 min. Then, 10 μL of the medium from the outer cell insert was collected and transferred to a well of 96-well plate containing 90 μL of phenol red-free culture medium. Fluorescence in each well was measured at 490 nm (excitation)/520 nm (emission).

### Histopathology of the pancreas

Tissue sectioning was done following a standard routine protocol of 4% formalin fixation, dehydration in conventional gradient alcohol, hyalinization with xylene, paraffin embedding, and tissue sections (4-μm). Tissue sections were stained with H&E. Histopathological changes in the pancreas tissue sections were observed under an optical microscope.

### Immunofluorescence microscopy

Cells treated with the appropriate agents were fixed in 4% paraformaldehyde in PBS for 15 min at room temperature. After rinsing in TD buffer (PBS with 1% dimethyl sulfoxide [DMSO] and 0.5% Triton X-100), the tissues were incubated in 2% bovine serum albumin (BSA) diluted in PBS for 30 min at room temperature to minimize nonspecific binding. Subsequently, the cells were incubated overnight at 4°C with primary antibodies against claudin 5 (Abcam, United Kingdom, 1:100), occludin (Abcam, United Kingdom, 1:200), ZO-1 (Abcam, United Kingdom, 1:500), and JAM-C (Santa Cruz, United States, 1:50) in a solution of 1% BSA in PBS. Following three washes with TD buffer, the cells were incubated with secondary antibodies (goat anti-rat or rat anti-goat IgG conjugated with Alexa Fluor 488, Jackson Immuno Research, United States, 1:100 in a mixture of 1% BSA in PBS) for 30 min at room temperature. Stained whole-mount cells were then mounted endothelial side up on a slide and counterstained with DAPI. Negative controls were included, consisting of cells without primary antibodies.

For preparation of paraffin sections, pancreas tissues were subjected to a series of dehydration steps in xylene-xylene-anhydrous-95% alcohol-75%, and alcohol-75% for 10 min each. After washing in PBS three times, the sections were subjected to antigen retrieval by boiling in citrate buffer (BOSTER, China) for 10 min. Following natural cooling, the sections were rinsed three times with PBS and incubated for 20 min at room temperature with primary antibodies against claudin 5, occludin, ZO-1, and JAM-C in a mixture of 1% BSA in PBS. The subsequent steps were consistent with those for cells. Finally, the sections and cultured cells were visualized using a laser confocal microscope (Olympus).

### qRT-PCR

Total RNA from HUVECs and tissues samples was extracted using the MiniBEST universal RNA Extraction Kit (9,796, TaKaRa Bio companies, Japan). A TAKARA *PrimeScript*™ RT Master Mix (RR036A, TaKaRa Bio companies, Japan) was used for reverse transcription, followed by qRT-PCR using a SYBR Green *Premix Ex Taq*™ (TliRNaseH Plus, RR420A, TaKaRa Bio companies, Japan) kit. The sequences of the primers used in this study are shown in Table 1.

**TABLE 1:** Sequences of primers used in this study.

Source	Gene	Forward and reverse sequences
Human	Claudin5	Forward 5′CAG​AGG​CAC​CAG​AAT​CAG​C3 ′
		Reverse 5′CCT​ACC​AGA​CAC​AGC​ACC​AG 3′
	Occludin	Forward 5′CAC​ACT​TGC​TTG​GGA​CAG​AG 3′
		Reverse 5′TAG​CCG​TAA​CCG​TAG​CCG​TA 3′
	ZO-1	Forward 5′AAC​CAT​CTT​TGG​ACC​GAT​TG 3′
		Reverse 5′GGT​CAG​TTC​CAG​CAT​CTC​GT 3′
	JAM-C	Forward 5′CAG​AGC​CAA​TCC​CAG​GTT​C 3′
		Reverse 5′CCC​AAT​AAT​CCC​AGC​AAT​GT 3′
	β-actin	Forward 5′CCT​GGC​ACC​CAG​CAC​AAT 3′
		Reverse 5′GGG​CCG​GAC​TCG​TCA​TAC 3′
Rat	Claudin5	Forward 5′CAG​AGG​CAC​CAG​AAT​CAG​C 3′
		Reverse 5′CCT​ACC​AGA​CAC​AGC​ACC​AG 3′
	Occludin	Forward 5′CAC​ACT​TGC​TTG​GGA​CAG​AG 3′
		Reverse 5′TAG​CCG​TAA​CCG​TAG​CCG​TA 3′
	ZO-1	Forward 5′AAC​CAT​CTT​TGG​ACC​GAT​TG 3′
		Reverse 5′GGT​CAG​TTC​CAG​CAT​CTC​GT 3′
	JAM-C	Forward 5′CAG​AGC​CAA​TCC​CAG​GTT​C 3′
		Revers e 5′CCC​AAT​AAT​CCC​AGC​AAT​GT 3′
	β-actin	Forward 5′TGT​CAC​CAA​CTG​GGA​CGA​TA 3′
		Reverse 5′GGG​GTG​TTG​AAG​GTC​TCA​AA 3′

### Western blot analysis

For Western blot analysis, both cells and pancreas tissues were lysed in RIPA lysis buffer (Beyotime Bio, China) supplemented with phenylmethanesulfonyl fluoride (PMSF, Beyotime Bio, United States). Equal amounts of protein from each sample were separated using SDS-PAGE electrophoresis, transferred onto a PVDF membrane (Beyotime Bio, China), and subsequently blocked with either 5% fat-free milk or 3% BSA in Tris-buffered saline with Tween 20 (TBST) at room temperature for 1 h. Following blocking, the membrane was incubated with the primary antibody, and the standard Western blotting procedure was carried out.

### Statistical analysis

SPSS 20.0 (Windows version) was used for statistical analysis. All data are expressed as the mean ± standard deviation (mean ± SD). One-way ANOVA was used to test differences between multiple sample means. The Student’s t-test was used to determine differences between two sample means (data in a normal distribution). The Mann-Whitney U (*Z*-value) test was used for data that were not normally distributed. If the variances were the same, a comparison between groups was performed using the LSD test (*F*-value). The Games-Howell method was used if the variances were not the same. *p* < 0.05 was set as statistical significance.

## Results

### Dose-dependent effect of DCQD on cell proliferation

**FIGURE 1:**
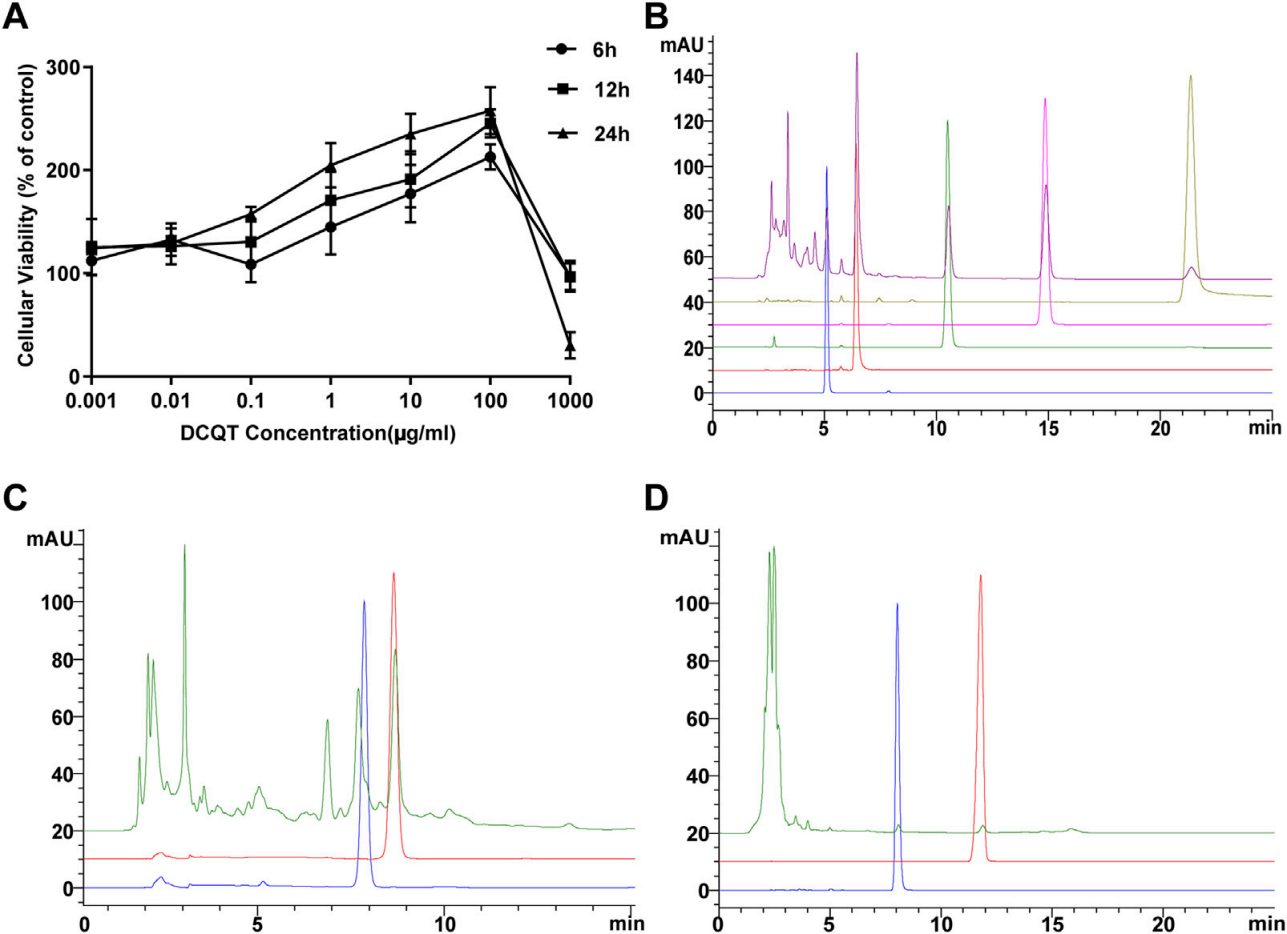
Effects of DCQD dosage and composition analysis. **(A)** Dose and time-dependent effects of DCQD: HUVEC viability increased at concentrations ranging from 1 ng/mL to 100 μg/mL. However, viability sharply declined when concentrations exceeded 100 μg/mL, with decreasing viability observed with longer incubation times. **(B)** HPLC analysis of *Rheum palmatum L*. components: Peaks of aloe-emodin, rhein, rheum emodin, chrysophanol, and physcion methyl were detected at 5.1, 6.4, 10.5, 14.9, and 21.4 min, respectively. No impurity interference was observed at the peaks of these five components. **(C)** HPLC analysis of *Magnolia henryi Dunn* components: Peaks of naringin and hesperidin were observed at 7.9 and 8.7 min, respectively. No impurity interference was detected at the peaks of these two components. **(D)** HPLC analysis of *Citrus aurantium L.* components: Peaks of magnolol and honokiol were identified at 11.8 and 8.0 min, respectively. No impurity interference was noted at the peaks of these two components. Please refer to the Materials and Methods section for detailed information on the HUVEC viability experiments and HPLC analysis.

As shown in Figure 1A, there was a dose-dependent effect of DCQD on cell viability (mean ± SD, *n* = 7 per experiment). Compared to the control group, the cell viability of the HUVECs increased with elevating concentrations of DCQD in the range of 1 ng/mL∼100 μg/mL but decreased when the DCQD dose exceeded 100 μg/mL. Cell proliferation nearly stopped in response to 1 mg/mL DCQD. We also observed that cell viability decreased with longer incubation time.

### Determination of DCQD components using HPLC analysis

**FIGURE 2:**
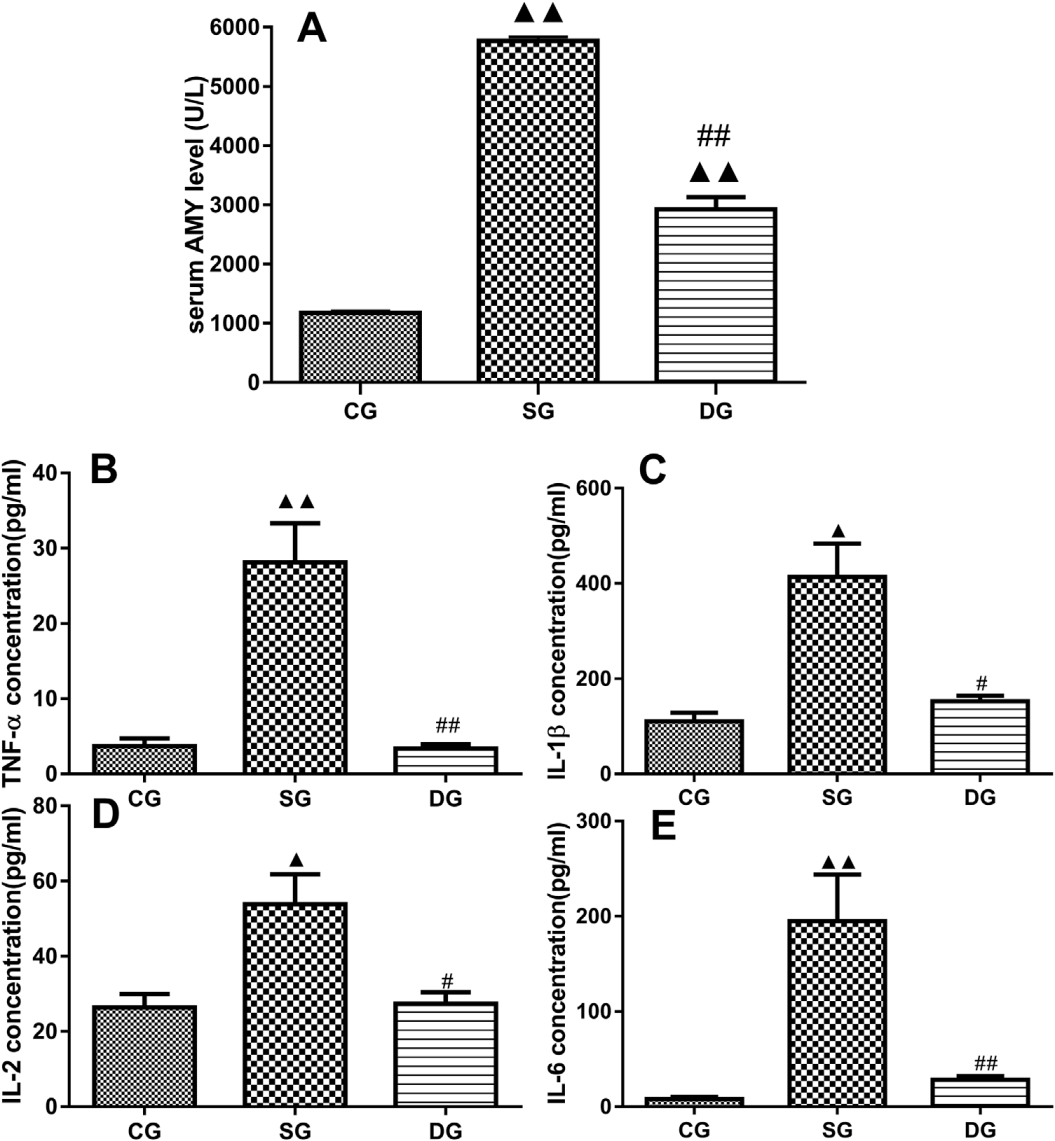
Serum levels of inflammatory markers in the treatment groups. Measurements of serum levels of Amy, TNF-α, IL-1β, IL-2, and IL-6 in different treatment groups. Levels of Amy **(A)**, TNF-α **(B)**, IL-1β **(C)**, IL-2 **(D)**, and IL-6 **(E)** were markedly elevated in the SG group but significantly decreased in the DG group. Statistical analysis revealed significant differences compared to the CG and SG groups; ^▲^ *p* < 0.05, ^▲▲^ *p* < 0.01, and ^#^ *p* < 0.05, ^##^i < 0.01, respectively. Please refer to the Materials and Methods section for detailed information on the measurements of serum levels of cytokines.

As shown in Figure 2, nine major components of DCQD were identified using HPLC analysis. The peaks of aloe-emodin, rhein, rheum emodin, chrysophanol, and physcion methyl ether appeared at 5.1, 6.4, 10.5, 14.9, and 21.4 min, respectively, and there was no impurity interference at the peaks of the five components in the samples (Figure 1B). The peaks of naringin and hesperidin appeared at 7.9 and 8.7 min, respectively, and there was no impurity interference at the peaks of the two components in the samples (Figure 1C). The peaks of magnolol and honokiol appeared at 11.8 and 8.0 min, respectively, and there was no impurity interference at the peaks of the two components in the samples (Figure 1D).

### Measurement of serum levels of Amy, TNF-α, IL-1β, IL-2, and IL-6

As shown in Figure 2, there were statistically significant differences in serum levels of Amy, TNF-α, IL-1β, IL-2, and IL-6 (mean ± SD, n = 10) between the SAP and DCQD groups. Serum levels of the above-mentioned cytokines were higher in the SAP groups compared to the control group (*p* < 0.01 for Amy, TNF-α, IL-6; *p* < 0.05 for IL-1β and IL-2). However, there were no significant difference in these cytokines between the DCQD group and CG group except for Amy (*p* < 0.01 for Amy; *p* > 0.05 for TNF-α, IL-6 IL-1β, and IL-2).

### Pathological changes in the pancreas

No obvious abnormalities were observed in the abdominal cavity of rats in the CG group, but there was ascites in the rats in the SAP group, with scattered saponified spots in their abdominal cavity. Seven rats in the SAP group survived. The pancreas size of rats in the SAP group was significantly enlarged, with obvious “jelly-like” necrotic foci on the surface. Three rats in the DG group had small amounts of ascites and a swollen pancreas with red necrotic foci, while the pancreas size in the other rats was similar to the rats in the normal group.

**FIGURE 3:**
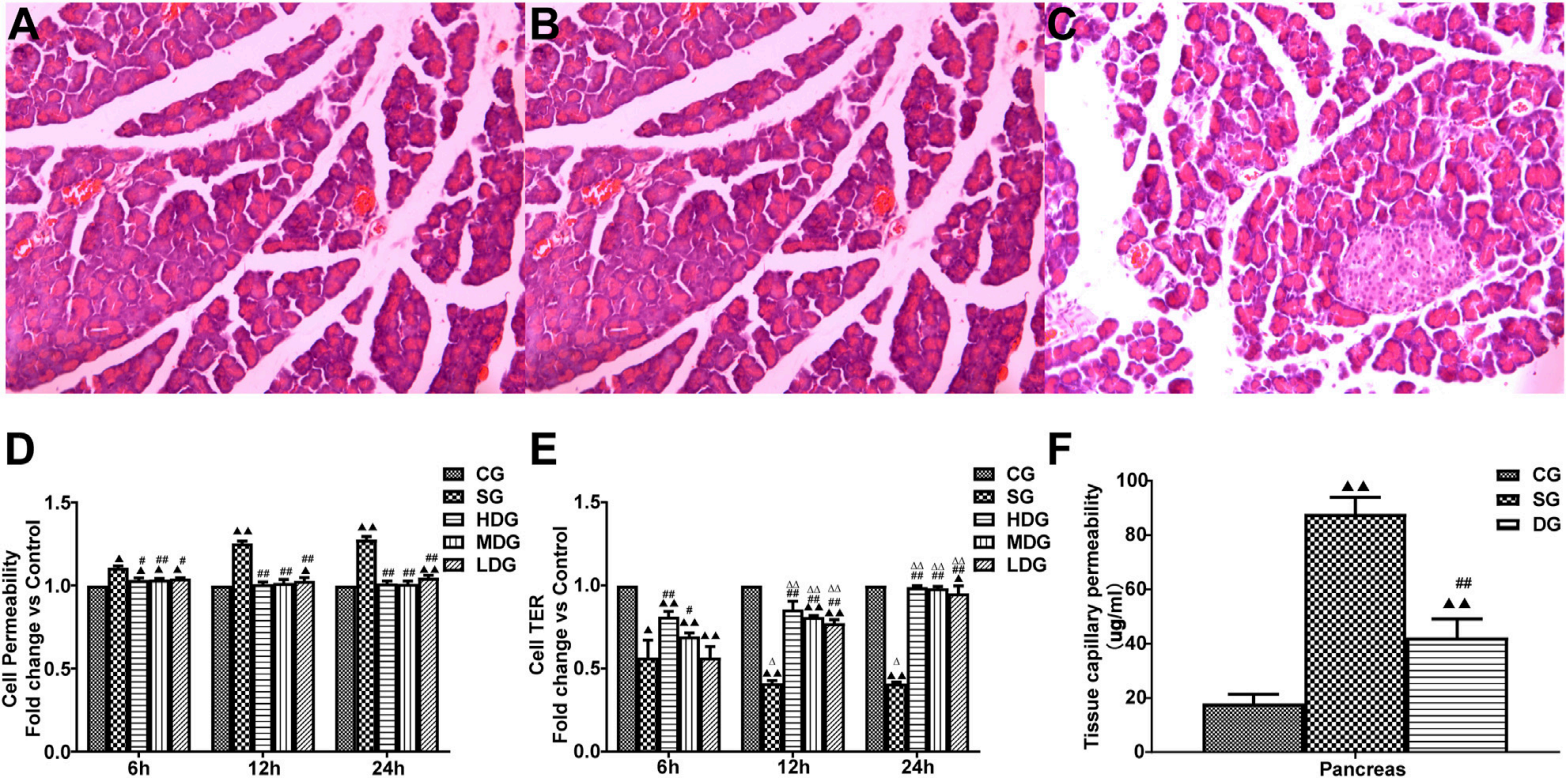
Protective effects of DCQD on pancreatic tissue integrity and capillary permeability in SAP. Histological changes in pancreatic tissue and the protective role of DCQD, as evidenced by H&E staining (×200). **(A)** Pancreatic ducts and acini appeared normal without obvious abnormalities in the CG group. **(B)** Marked destruction of pancreatic tissue, including disordered lobular structure, interstitial hyperemia, hemorrhage, and areas of coagulation necrosis with inflammatory cell infiltration along the necrotic pancreas in the SG group. **(C)** Mild pathological changes observed in the DG groups compared to the SG groups. Permeability in the pancreas and HUVECs increased, while the TER of the HUVECs decreased in the SAP treated groups. DCQD exhibited varying protective effects depending on dose and time. **(D)** Comparison of HUVEC permeability in different treatment groups. **(E)** Comparison of TER values in different treatment groups. **(F)** Effect of DCQD on pancreatic capillary permeability. Statistical analysis revealed significant differences compared to the CG and SG groups, and the 6-h time point; ^▲^ *p* < 0.05, ^▲▲^ *p* < 0.01, ^#^ *p* < 0.05, ^##^ *p* < 0.01, ^△^ *p* < 0.05, ^△△^ *p* < 0.05, respectively. Please refer to the Materials and Methods section for detailed information on the examination of the histological changes in pancreatic tissue.

H&E staining results showed a clear pancreatic lobule structure of rats in the CG group. There was no obvious abnormality in the pancreatic ducts and acini. In the SG group, there were structural disorders in the pancreas, such as indistinct lobular structure, interstitial hyperemia, and hemorrhage, and coagulation necrosis. In addition, there was an increase in infiltrating inflammatory cells in the necrotic pancreas and interstitial small blood. However, the pathological changes in the pancreas were remarkably less in the DG group compared to the SG group (Figures 3A–C).

### TER and cell permeability

The TER value (mean ± SD, *n* = 8) in the SG or low-dose DCQD group was higher than that in the CG group. However, the TER value after 12 h of HDG or 2 or 4 h of MDG was similar to that in the CG group. Compared to the SAP group, TER in both the HDG and MDG groups increased significantly. There was no statistical difference in TER between the LDG and SG groups at 6 h, but TER in the LDG group significantly increased at 12 h (Figures 3D, E).

### Value of pancreatic tissue capillary permeability

Tissue capillary permeability (mean ± SD, *n* = 10) in the pancreas was higher in both the SG and DG groups compared to the CG group (*F* = 36.521; *p* < 0.00, *p* < 0.001 for all groups), but permeability in the pancreas was lower in the DG group compared to the SG group (Figure 3F).

### Cell and pancreatic tissue mRNA levels of TJ-associated genes

#### mRNA levels of TJ-associated genes were determined using qRT-PCR

**FIGURE 4:**
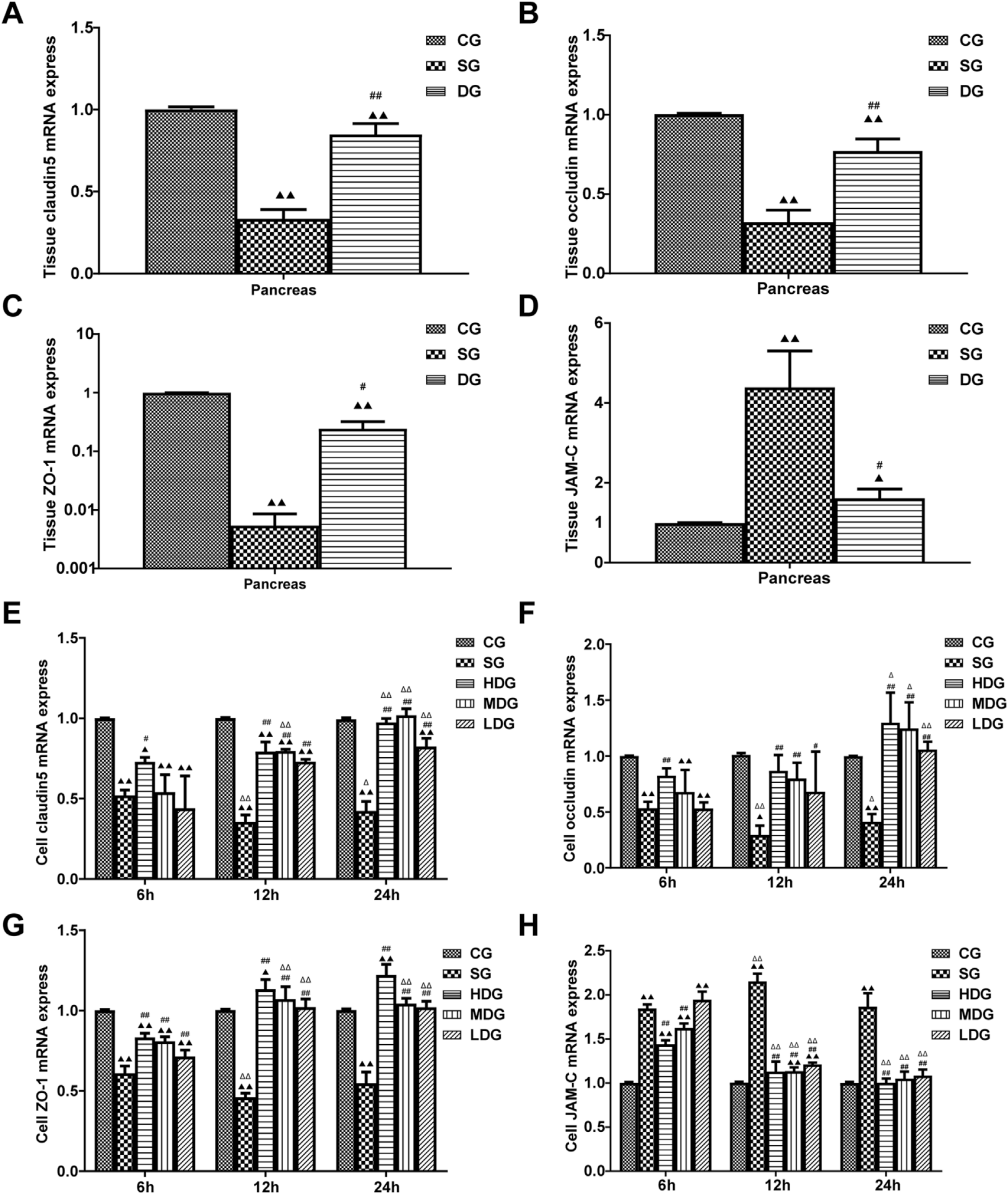
Impact of DCQD on TJ protein expression in HUVECs and pancreatic tissue during SAP. Alterations in TJ protein expression in HUVECs and pancreatic tissue, along with the time- and dose-dependent protective effects of DCQD. **(A)** Changes in mRNA levels of claudin5 genes in pancreatic tissue. **(B)** Changes in mRNA levels of occludin genes in pancreatic tissue. **(C)** Changes in mRNA levels of ZO-1 genes in pancreatic tissue. **(D)** Changes in mRNA levels of JAM-C genes in pancreatic tissue. **(E)** Changes in mRNA level of claudin five genes in HUVECs. **(F)** Changes in mRNA levels of occludin genes in HUVECs. **(G)** Changes in mRNA levels of ZO-1 genes in HUVECs. **(H)** Changes in mRNA levels of JAM-C genes in HUVECs. Claudin5, occludin, and ZO-1 expression decreased in both HUVECs and pancreatic tissue, while JAM-C expression sharply increased in the SG group. DCQD’s protective effect appeared to be dose- and time-dependent. Statistical significance compared to the CG and SG groups, and the 6-h time point;^▲^ *p* < 0.05, ^▲▲^ *p* < 0.01, ^#^ *p* < 0.05, ^##^ *p* < 0.01, ^△^ *p* < 0.05, ^△△^ *p* < 0.05, respectively. Please refer to the Materials and Methods section for detailed information on the methods for detecting TJ protein expression.

*In vivo*, the pancreatic mRNA levels (mean ± SD, *n* = 5) of claudin 5, occludin, and ZO-1 were decreased in the DG group, while JAM-C levels were increased compared to the CG group. mRNA levels of claudin 5, occludin, and ZO-1 in the pancreas were upregulated in the DG groups, but JAM-C levels were slightly decreased compared to the SG group (Figures 4A–D).

*In vitro*, the mRNA levels of claudin 5, occludin, and ZO-1 (mean ± SD, *n* = 4) decreased in the SG group during the period from 6 h to 24 h after incubation, and reached the lowest level at 12 h, while the levels of claudin 5, occludin, and ZO-1 were upregulated in the DG group compared to the CG group. We observed more changes in mRNA levels of claudin 5, occludin, and ZO-1 with higher treatment concentrations and longer incubation times. In contrast, JAM-C mRNA levels (mean ± SD, *n* = 4) were upregulated in the SG group from 6 h, peaking at 12 h, and decreasing slightly at 24 h. JAM-C mRNA levels were downregulated by DG treatment in a dose-time-dependent manner and decreased with higher treatment concentrations and longer incubation times (Figures 4E–H).

### Cell and tissue expression levels of TJ-associated proteins

**FIGURE 5:**
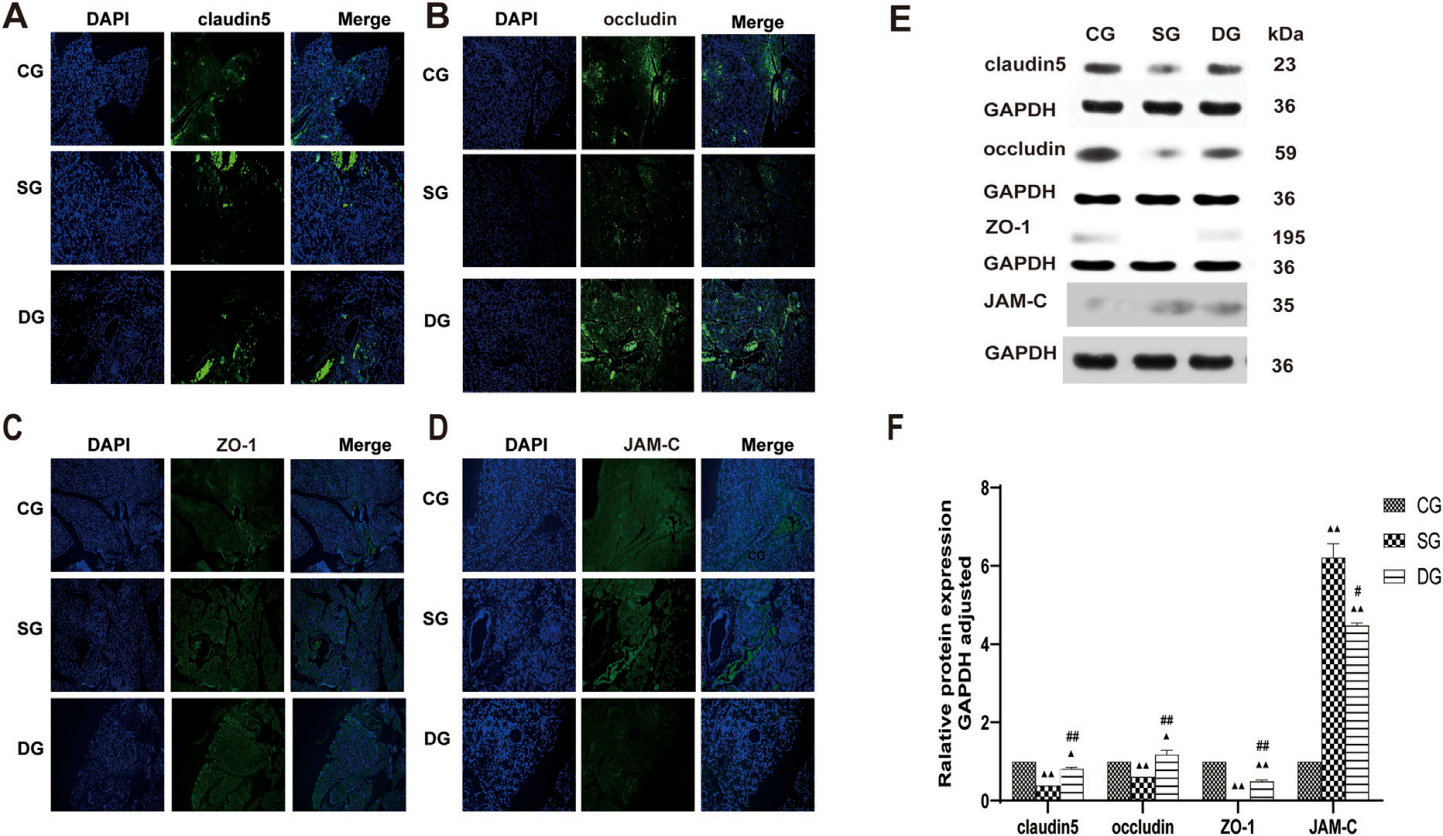
DCQD restores TJ-associated protein expression in pancreatic tissue affected by SAP. Restoration of TJ-associated proteins in the pancreas induced by SAP after DCQD treatment. **(A)** Immunofluorescence of claudin five expression in pancreatic tissue. **(B)** Immunofluorescence of occludin expression in pancreatic tissue. **(C)** Immunofluorescence of ZO-1 expression in pancreatic tissue. **(D)** Immunofluorescence of JAM-C expression in pancreatic tissue. **(E)** Western blot analyses of TJ-associated proteins in pancreatic tissue, with GAPDH serving as the internal loading control. **(F)** Analyses of TJ-associated protein expression in pancreatic tissue. Immunofluorescence and protein expression of claudin 5, occludin, and ZO-1 were significantly downregulated, while JAM-C expression was increased in the SG group. DCQD treatment upregulated expression of claudin 5, occludin, and ZO-1 and downregulated JAM-C expression in the pancreas. Statistical significance compared to the CG and SG groups; ^▲^ *p* < 0.05, ^▲▲^ *p* < 0.01, ^#^ *p* < 0.05, ^##^ *p* < 0.01, respectively. Please refer to the Materials and Methods section for detailed information on the methods for detecting THJ protein expression.

Cell and tissue expression of TJ-associated proteins were analyzed both *in vivo* and *in vitro*. *In vivo*, claudin5, occludin, and ZO-1 fluorescence intensity showed an even distribution in the CG group, while it was weakened in the SG group, with a notable aggregation of fluorescence observed in the blood vessels. Conversely, pancreatic fluorescence was enhanced in the DG group compared to the SG group, with strong fluorescence signals observed in the blood vessels (Figures 5A–C). JAM-C fluorescence intensity displayed a uniform distribution in the CG group, with stronger fluorescence observed in the blood vessels. In the SG group, JAM-C fluorescence was significantly enhanced, with some leakage from necrotic pancreatic tissues and increased fluorescence in the blood vessels. In the DG group, fluorescence levels nearly returned to those observed in the CG group (Figure 5D). The protein expression of claudin 5, occludin, and ZO-1 (mean ± SD, *n* = 5) was decreased in the DG group compared to the CG group, whereas JAM-C expression was increased. Conversely, in pancreatic tissues, the expression of claudin 5, occludin, and ZO-1 was significantly upregulated in the DG groups, with a slight decrease in JAM-C expression compared to the SG group (Figures 5E, F).

**FIGURE 6:**
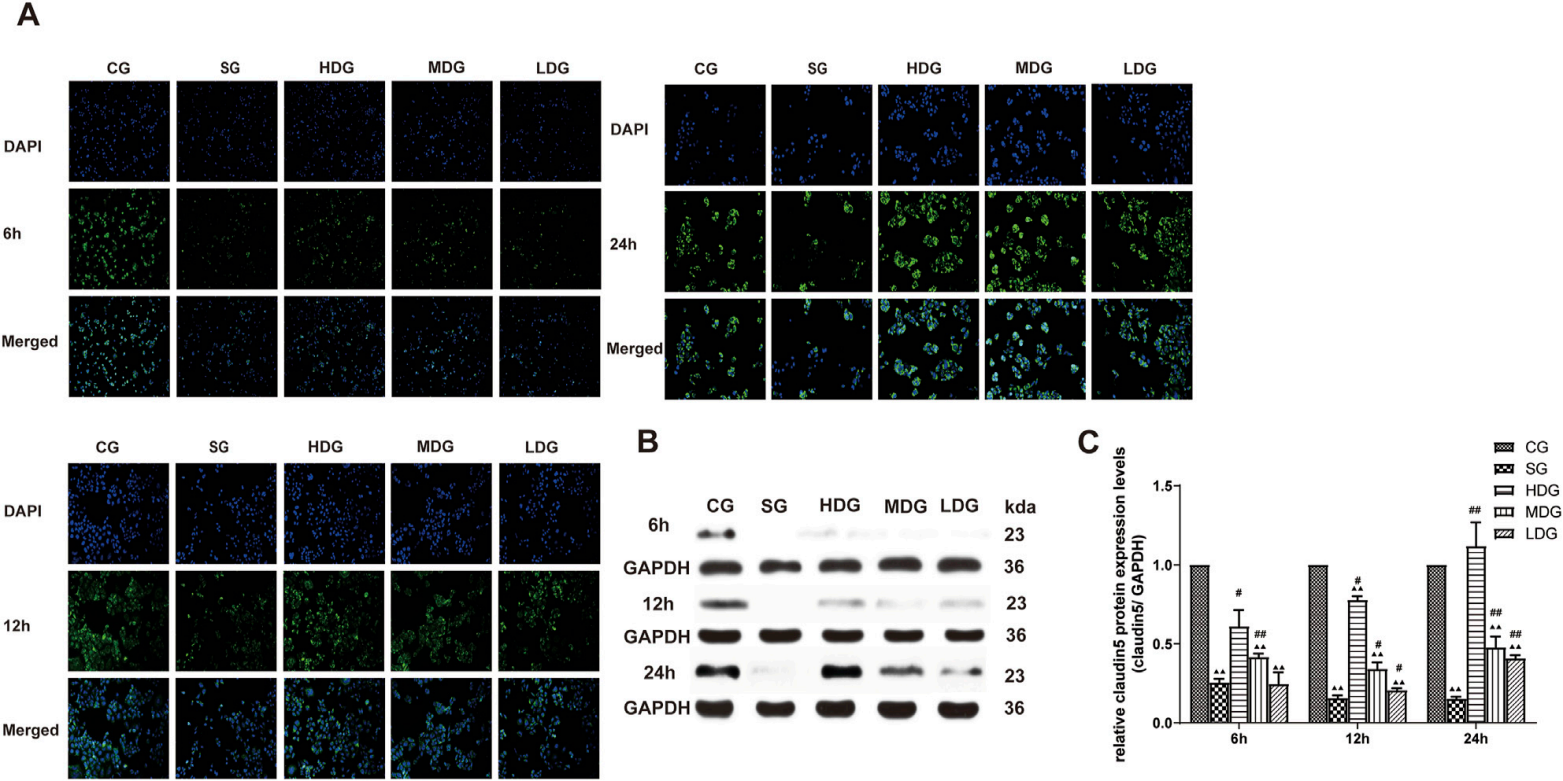
DCQD reverses theh decline in claudin five expression induced by SAP *in vitro*. Restoration of claudin five expression *in vitro* following treatment with DCQD after SAP. **(A)** Immunofluorescence images of claudin five at different time points *in vitro*. **(B)** Western blot analyses of claudin five at different time points *in vitro*, with GAPDH serving as the internal loading control. **(C)** Analyses of claudin five expression at different time points *in vitro*. Fluorescence intensity and protein expression of claudin five were significantly weaker in the SG group compared to the CG and tended to decrease with prolonged modeling time. High doses of DCQD effectively upregulated claudin five expression, demonstrating a time correlation, while the effect was less pronounced in MDG and LDG groups. Statistical significance compared to the CG and SG groups; ^▲^ *p* < 0.05, ^▲▲^ *p* < 0.01, ^#^ *p* < 0.05, ^##^ *p* < 0.01, respectively. Please refer to the Materials and Methods section for detailed information on the methods for detecting claudin5 expression.

**FIGURE 7:**
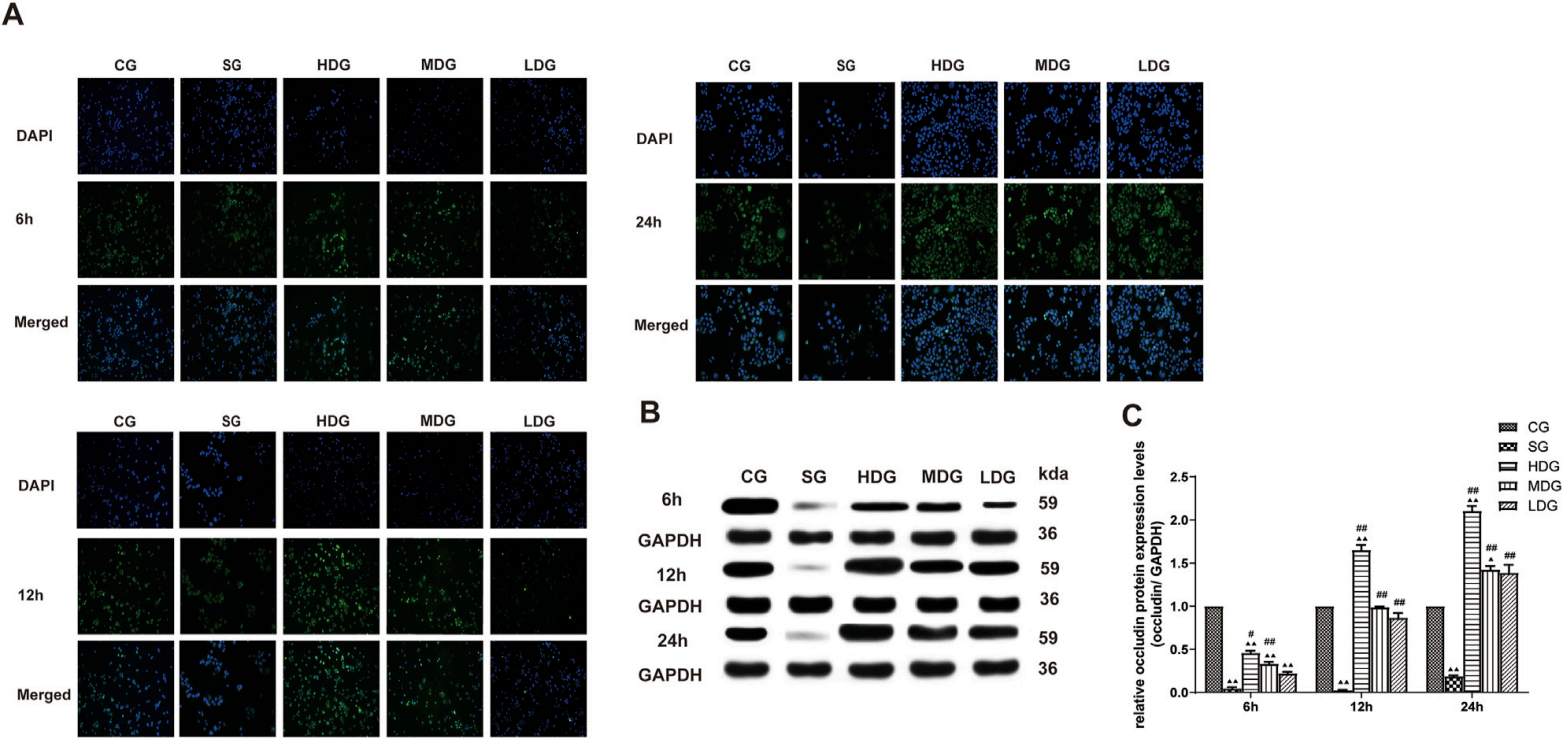
DCQD counteracts the reduction in occludin induced by SAP *in vitro*. Restoration of occludin expression *in vitro* following treatment with DCQD SAP. **(A)** Immunofluorescence images of occludin at different time points *in vitro*. **(B)** Western blot analyses of occludin expression at different time points *in vitro*, with GAPDH serving as the internal loading control. **(C)** Analyses of occludin expression at different time points *in vitro*. Fluorescence intensity and protein expression of occludin were significantly weaker in the SG group compared to the CG and tended to decrease with prolonged modeling time. DCQD treatment effectively upregulated occludin expression, exhibiting a partly dose-time correlation, although the effect was less pronounced in the LDG group. Statistical significance compared to the CG and SG groups; ^▲^ *p* < 0.05, ^▲▲^ *p* < 0.01, ^#^ *p* < 0.05, ^##^ *p* < 0.01, respectively. Please refer to the Materials and Methods section for detailed information on the methods for detecting occludin expression.

**FIGURE 8:**
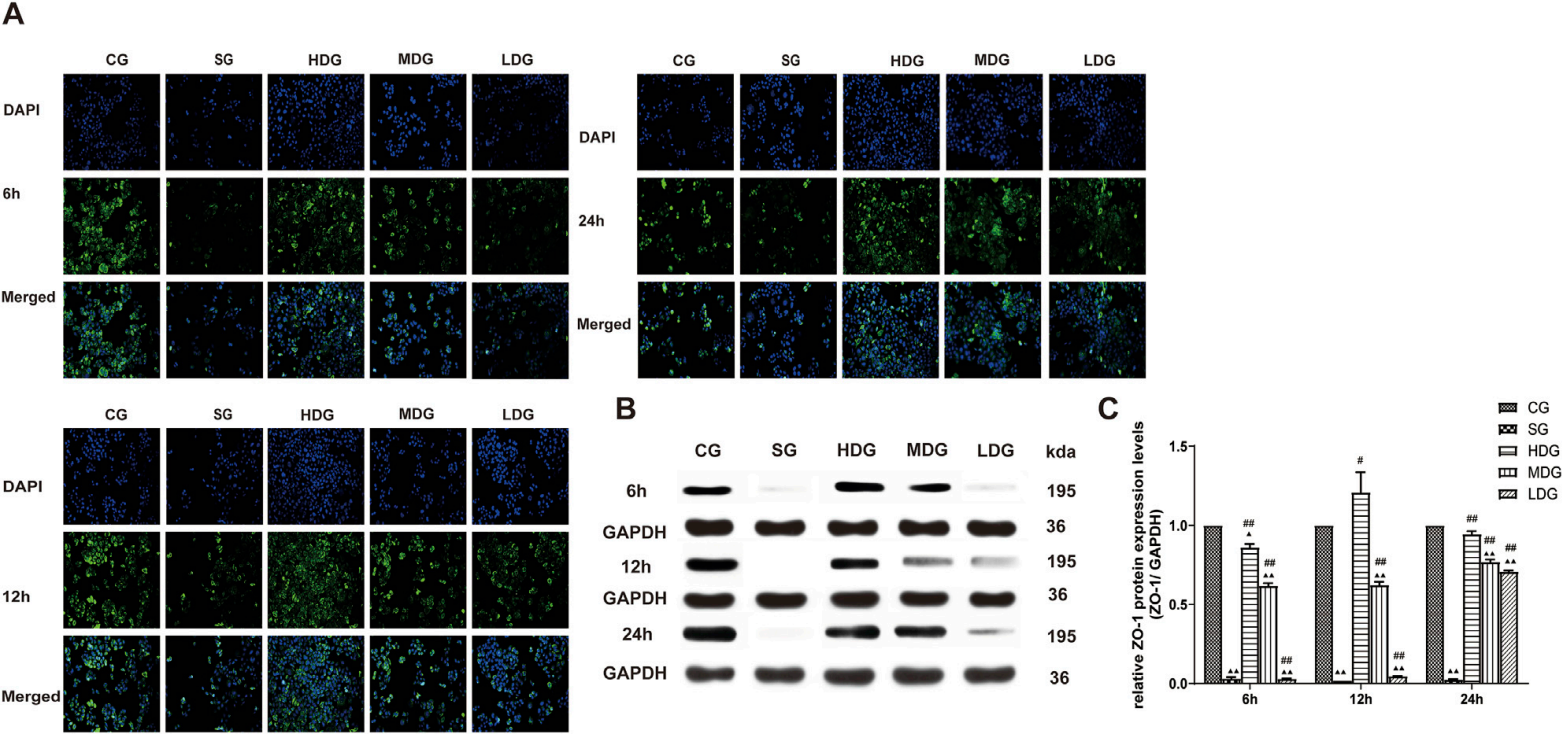
DCQD reverses the decrease in ZO-1 expression induced by SAP *in vitro*. Reversal of ZO-1 expression *in vitro* following treatment with DCQD after SAP. **(A)** Immunofluorescence images of ZO-1 at different time points *in vitro*. **(B)** Western blot analyses of ZO-1 at different time points *in vitro*, with GAPDH serving as the internal loading control. **(C)** Analyses of ZO-1 expression at different time points *in vitro*. Fluorescence intensity and protein expression of ZO-1 were weaker in the SG groups compared to the CG and tended to decrease with prolonged modeling time. Conversely, DCQD treatment effectively upregulated ZO-1 expression, exhibiting a partly dose-time correlation, although the effect was less pronounced in the LDG group. Statistical significance compared to the CG and SG groups; ^▲^ *p* < 0.05, ^▲▲^ *p* < 0.01, ^#^ *p* < 0.05, ^##^ *p* < 0.01, respectively. Please refer to the Materials and Methods section for detailed information on the methods for detecting ZO-1 expression.

**FIGURE 9:**
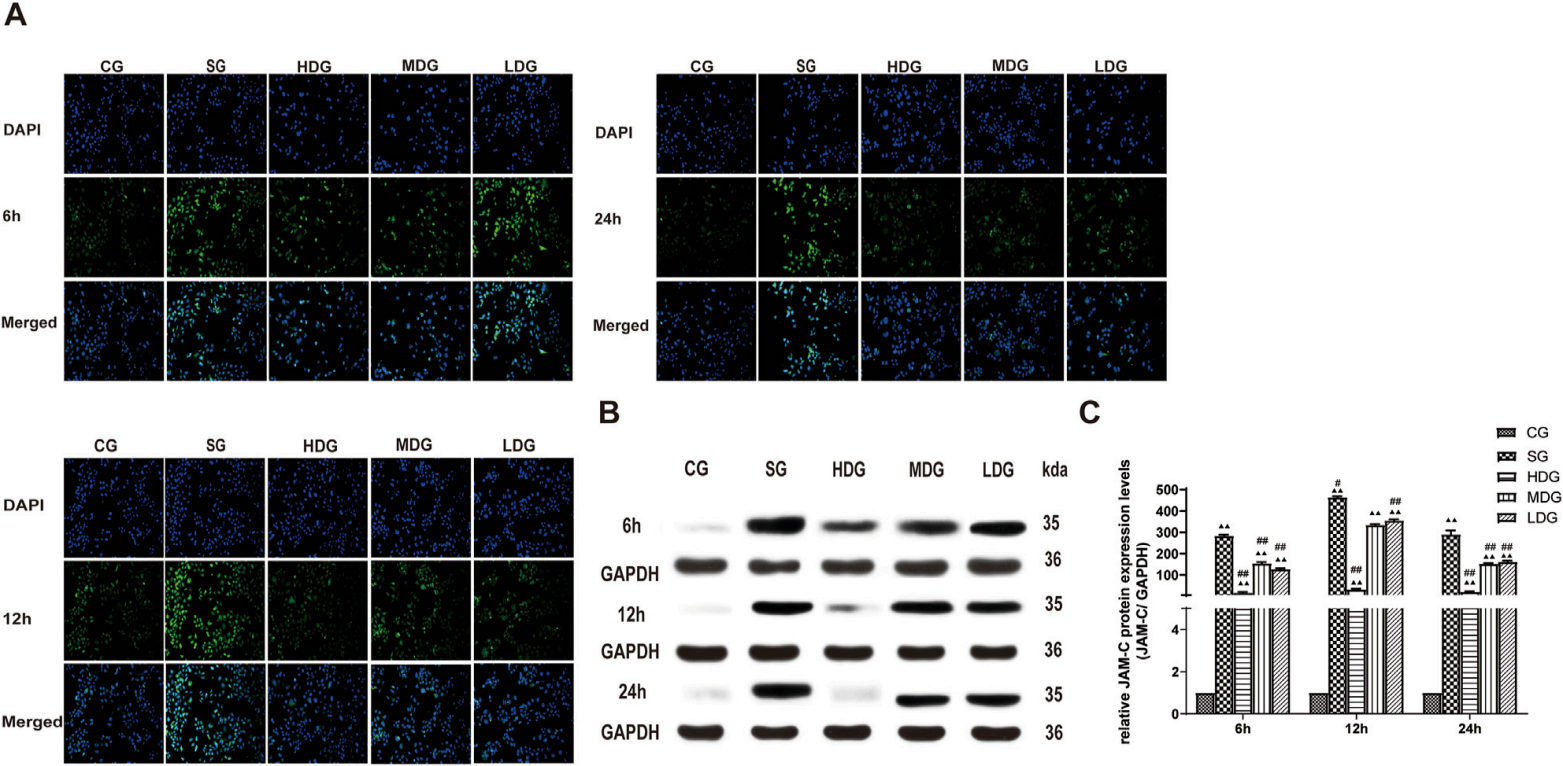
DCQD attenuates the elevation of JAM-C expression induced by SAP *in vitro*. Attenuation of JAM-C expression *in vitro* following treatment with DCQD after SAP. **(A)** Immunofluorescence images of JAM-C at different time points *in vitro*. **(B)** Western blot analyses of JAM-C at different time points *in vitro*, with GAPDH serving as the internal loading control. **(C)** Analyses of JAM-C expression at different time points *in vitro*. Fluorescence intensity and protein expression of JAM-C were increased in the SG group compared to the CG. However, DCQD treatment effectively downregulated JAM-C expression, with a more pronounced effect observed with higher doses and longer treatment durations. Statistical significance compared to the CG and SG is groups; ^▲^ *p* < 0.05, ^▲▲^ *p <* 0.01, ^#^ *p* < 0.05, ^##^ *p* < 0.01, respectively. Please refer to the Materials and Methods section for detailed information on the methods for detecting JAM-C expression.

*In vitro,* the immunofluorescence of claudin 5 (Figure 6A), occludin (Figure 7A), and ZO-1 (Figure 8A) in the SG group as significantly attenuated, while expression of these proteins was regulated to different degrees in a time-dependent manner in the HDG group. Moderate-dose and low-dose treatments did not show significant effects initially but fluorescence signals were enhanced over time. Conversely, JAM-C was strongly expressed in the SG group. High-dose and moderate-dose treatments inhibited JAM-C expression to varying degrees in a time-dependent manner, while the effect of the low-dose treatment was not prominent (Figure 9A).

The protein expression of claudin 5 (Figures 6B, C), occludin (Figures 7B, C), and ZO-1 (Figures 8B, C) (mean ± SD, *n* = 4 per experiment) decreased in the SG group from 6 h, reaching the lowest levels at 12 h, while in the DG group, expression of these proteins was upregulated compared to the CG group. Variations in protein expression of claudin 5, occludin, and ZO-1 was more pronounced with higher treatment concentrations and longer incubation times. Conversely, JAM-C expression (mean ± SD, *n* = 4 per experiment) was upregulated in the SG group from 6 h, peaking at 12 h, and slightly decreasing at 24 h. However, DG treatment resulted in a dose-time-dependent downregulation of JAM-C expression, which decreased with higher treatment concentrations and longer incubation times (Figures 9B, C).

## Discussion

SAP patients often die early from concurrent SIRS and MODS. When acute pancreatitis occurs, a large number of inflammatory factors and toxic substances enter the microcirculation, triggering SIRS, which not only causes MODS but also damages vascular endothelial cells and the integrity of capillary endothelial barrier function, leading to CLS. When vascular permeability increases, the endothelial barrier function can be severely impaired, causing vascular contents to leak, leading to an increase in vasospasm, blood stasis, tissue ischemia, and necrosis (Garg and Singh, 2019). Severe capillary leakage is also an important risk factor in SAP complicated by MODS (Sukriti et al., 2014). Therefore, prevention of capillary leakage is critical to halt disease processes and reduce SAP-associated morbidity and mortality.

To investigate endothelial cell damage caused by SAP, we used an *in vitro* model of SAP established by incubating HUVECs with serum from rats with induced pancreatitis (Zhong, 2010). *In vitro* endothelial cell barrier function can be monitored by measuring the TER of a single layer of endothelial cells in a fused state. A higher TER value indicates the existence of a stronger barrier and lower cell permeability. The permeability of the endothelial cell barrier to macromolecular substances is an important indicator of endothelial cell barrier integrity. In this experiment, FITC-Dextran (relative molecular weight 40,000 Da) was used as a tool to estimate barrier function in a monolayer of cultured HUVECs. The lower permeation rate (i.e., weaker fluorescence intensity) of FITC-Dextran indicated optimal barrier function of the HUVECs. Results of the *in vitro* SAP model of endothelial damage showed that in the early stage of SAP, the TER of HUVECs decreased and their fluorescence transmission rate increased (6 h) compared to that in the normal group. The pancreatic permeability also was increased with increased incubation time of the rat serum (Figure 3F), suggesting that the endothelial barrier was compromised at the early stage of SAP. This result is consistent with the existing theory that the capillary endothelial barrier is present at the early stage of SAP.

We next sought to determine the effect of DCQD in SAP. The lyophilized powder of DCQD was prepared and HPLC was used to determine the drug components. There were no impurities in the DCQD preparation and the content of the lyophilized powder remained stable for 108 h. The effect of DCQD on HUVEC viability was determined using the CCK-8 assay. When its concentration ranged from 0.001 to 100 μg/mL, DCQD promoted rapid proliferation of HUVECs in a time-dependent manner (Figure 1A). Therefore, 100 μg/mL was selected as the highest concentration of DCQD used in further experiments. Based on a report by Yikui et al. (Li, 1999), half of the highest concentration (50 μg/mL) was used as the middle dose concentration, and a quarter of the highest concentration (25 μg/mL) was used as the low dose concentration. TER values of the HUVECs were determined after incubation with high and medium doses of DCQD. The high and medium doses of DCQD increased cell permeability after 6 h of treatment. By 12 h, the permeability of the DCQD-treated HUVECs approximated that of the control, but there was a detectable difference at 24 h. The inhibitory effect of DCQD on capillary permeability was enhanced with increased incubation time. However, the inhibitory effect of DCQD on capillary permeability in the DMG group was less effective than that in the DHG group. There was no obvious inhibitory effect of DCQD in the LDG group at the early stage of incubation, but as the incubation time was prolonged, the cell permeability was partially attenuated by DCQD, suggesting that the inhibitory effect of DCQD on capillary permeability is dose- and time-dependent (Figures 3D, E).

Our study found that after 12 h of SAP induction, the EB exudation from pancreatic tissues increased, suggesting that the capillary permeability in the pancreas was elevated (Figure 3F). Serum levels of Amy and inflammatory factors (TNF-α, IL-1β, IL-2, and IL-6) were also significantly elevated, suggesting the release of large amounts of harmful inflammatory factors into the blood during SAP (Figures 2A–E). We also observed that the pancreatic tissue in the SAP group was damaged, with a large number of inflammatory cells and a diffuse distribution of erythrocytes in the intercellular space. These data indicate that vascular permeability was altered at the early stage of SAP.

Inter-endothelial cell junctions, the basement membrane, and the endothelium itself are key elements that determine the degree of endothelial permeability. TJs are located at the outermost part of the intercellular junction and act as a “gatekeeper” (Otani and Furuse, 2020). TJs are composed of tight junctional complexes formed by transmembrane proteins, including claudins, occludins, JAMs, and ZOs, all of which are arranged in a continuous anastomosis to form a meshwork structure that tightly connects endothelial cells. ZO-1 was the first identified TJ-associated protein (Vermette et al., 2018). Dervenis et al. (Dervenis et al., 2003) found that the molecular mechanisms underlying capillary leakage, ischemia, and necrosis of the intestinal mucosa caused by SAP onset were associated with ZO-1 downregulation and degradation of ZO-1 protein in the intestinal epithelium, resulting in poorly connected intestinal epithelial cells and intestinal barrier dysfunction. It has been reported that (Youakim and Ahdieh, 1999) TNF-α can reduce the barrier function of intestinal epithelial cells by regulating ZO-1 expression, rather than ZO-2, suggesting that ZO-1 plays an important role in the maintenance of TJs. Claudins alter paracellular permeability by directly regulating the switching of TJ transport channels (Krug et al., 2014) (PTJC). The expression of claudin 1, claudin 4, and claudin 15 in monolayer epithelial cells was positively correlated with TER, whereas the expression of claudin two in high-resistance type I canine kidney cells was negatively correlated with TER (Oliveira and Morgado-Díaz, 2007). Claudin five is often used as a biomarker of barrier function in various inflammatory diseases. For example, acute lung injury is accompanied by decreased expression of claudin five in the lung epithelial cells (Armstrong et al., 2012). Schreibelt et al. (2007) found that claudin five was only expressed in endothelial cells, however, its role in the regulation of capillary leakage in pancreatitis remains elusive. Occludin plays a key role in the maintenance of the TJ barrier and defense functions, but changes in its expression do not affect the structure of TJs. A dramatic decrease in occludin gene expression was detected at 24 h after onset of SAP in a mouse model with sparse, detached small intestinal epithelial microvilli (Wu et al., 2011). Loss and degradation of occludin protein might cause disruption of intestinal epithelial cell junctions and damage to the intestinal epithelial barrier. However, the effect of occludin on the regulation of capillary leakage in pancreatitis has not been reported. The intracellular PDZ-binding structural domain of JAMs is associated with other TJ-associated proteins (Mandell and Parkos, 2005). There are three JAM isoforms: JAM-A, B, and C. Mandell and Parkos (2005) found that JAM-C is mainly located in microvascular endothelial cells, which can be rapidly and transiently redistributed to intercellular junctions in response to stimulation with vascular endothelial growth factor or histamine during inflammation. In our previous study (Tian et al., 2013), we reported that increased vascular permeability and disease exacerbation were accompanied by upregulation of JAM-C in pancreatic tissue.

In the present study, changes in the expression of claudin 5, occludin, ZO-1, and JAM-C were determined at different time points after SAP induction, and changes in endothelial cell permeability were evaluated using TER and FITC-dextran assays. We observed that expression of claudin 5, occludin, and ZO-1 decreased gradually and JAM-C expression increased following SAP. We also observed a decrease in TER and an increase in permeability at 6, 12, and 24 h after modeling, suggesting that downregulated expression of TJ-associated genes at the early stage of SAP onset may occur in endothelial cells (Figure 3E). In response to inflammatory factors, cytoplasmic JAM-C transiently reaches the TJ early in the pathogenesis of SAP (Mandell and Parkos, 2005). It is noteworthy that the expression of claudin 5, occludin, and ZO-1 were lowest at 12 h after SAP incubation and slightly increased at 24 h (Figures 6–8). JAM-C expression also peaked at 12 h, indicating a rapid progression of SAP and severe capillary leakage within 24 h (Figure 9). We speculated that the inflammatory factors in SAP serum may reduce the expression of claudin 5, occludin, and ZO-1 and destroy the structural integrity of the TJ complex, disrupting the connection between endothelial cells and leading to an increase in vascular permeability. Thus, the increase in permeability results in vascular leak that aggravates tissue edema, ischemia, and necrosis.

JAM-C, as an adhesion molecule, can be temporarily redistributed to the TJ in response to inflammatory factors. Overexpression of JAM-C promotes leukocyte adherence, leading to excessive leukocyte activation and aggravated pancreatitis. We found that treatment with a high dose of DCQD downregulated JAM-C expression and maintained the integrity of the TJ. The protective effect of DCQD started from 6 h after incubation and became more effective with increased incubation time. The protective effect of the medium dose of DCQD also started from 6 h after incubation and gradually increased until 12 h. However, the protective effect of DCQD could not be detected in the low-dose group at 6 h, suggesting the protective effect of DCQD is dose- and time-dependent and is associated with regulation TJ-associated proteins.

Our study reveals that endothelial cell proteins claudin5, occludin, and ZO-1 undergo degradation, while intracytoplasmic JAM-C transiently localizes to the TJs during the early stage of SAP onset when stimulated by inflammatory factors. Our findings also verified that the rapid progression and severe capillary leakage in the first 24 h is a critical period in the treatment of SAP.

We hypothesize that inflammatory factors in SAP contribute to the degradation of claudin 5, occludin, and ZO-1 proteins, compromising the structural integrity of the TJ complex. Consequently, endothelial cells become less tightly connected, leading to elevated vascular permeability and significant leakage of vascular contents across the TJ. This exacerbates tissue edema, ischemia, and necrosis. Furthermore, JAM-C, which participates in TJ composition and belongs to the adhesion molecule family, undergoes temporary redistribution to the TJ in response to inflammatory factors during pancreatitis. The heightened expression of JAM-C promotes leukocyte adhesion to the TJ, triggering leukocyte over activation and exacerbating pancreatitis severity.

Our findings suggest that the herbal formula DCQD effectively improves SAP and decreases pancreatic vascular permeability by enhancing the expression of TJ-associated proteins claudin5, occludin, and ZO-1, while downregulating JAM-C expression. This action helps maintain TJ structure integrity between endothelial cells, preventing leakage of vascular contents, reducing tissue edema, enhancing vascular microcirculation, and ultimately averting capillary leakage during the early stages of SAP.

The evolutionary pattern and disease course of SAP can be better understood using Traditional Chinese Medicine (TCM) theory and the staging and dialectical theory proposed by Jiang Junming, who summarized the TCM methods included in the classic book “Yi huo Qing xia” (Xia et al., 2006). Modern pharmacology also postulates that the mechanisms underlying DCQD treatment involve the following aspects: inhibiting secretion of pancreatic enzymes, enhancing movement, restoration, and microcirculation in the gastrointestinal tract, reducing absorption of toxins in the intestine, preventing displacement of bacteria, inhibiting systemic inflammation, and promoting apoptosis of pancreatic cells.

In conclusion, DCQD treatment can improve capillary permeability at an early stage of SAP, and the protective effect of DCQD on capillary leakage and regulation of TJ-associated genes in the progress of SAP is time- and dose-dependent. The underlying mechanisms of DCQD-mediated protection may be related to regulation of endothelial cell TJ-associated genes. In future studies, we plan to individually examine the components of DCQD to determine which ingredient offers the most benefit to patients with acute pancreatitis, thereby promoting its broader clinical use.

## Data Availability

The raw data supporting the conclusion of this article will be made available by the authors, without undue reservation.

## Abbreviations

AP, acute pancreatitis; CG, Control group; CLS, capillary leakage syndrome; DCQD, Da-Cheng-Qi-decoction; FITC, dextran: fluorescein isothiocyanate-dextran; HPLC, high-performance liquid chromatography; HDG, high DCQD dose group; HUVECs, human umbilical vein endothelial cells; IL-1β, interleukin-1β; IL-2, interleukin-2; IL-6, interleukin-6; LDG, low DCQD dose group; MDG, medium DCQD dose group; qRT-PCR, quantitative teal-time polymerase chain reaction; SAP, severe acute pancreatitis; SG, SAP serum-treated model group/SAP group; SIRS, systemic inflammatory response syndrome; TCM, Traditional Chinese Medicine; TER, transendothelial electrical resistance; TNF-α, tumor necrosis factor α.
